# Supplementary material for: Chromothripsis during telomere crisis is independent of NHEJ, and consistent with a replicative origin
Source: Genome Res. 2019 May;29(5):737–49. doi: 10.1101/gr.240705.118 (PMC6499312; doi:10.1101/gr.240705.118)
Supplement: Supplemental Material [file supp_gr.240705.118_Supplemental_file_1.zip › contigs/annotated_contigs/DB80/contig.2.DB80_length_652_mean_cov_11.273006135.docx]

**DB80_length_652_mean_cov_11.273006135**

```
CGCCCGGCCAGACACAGTGTTCTTCATGTGAGGTAGAAATAACACATGCCTTTAAGGACTGCAGAATGATGAGTGGTGATGTTCTTGAA
 >chr3:6926575-6926836 + E=3e-139
CTCTTCCAATACTCCACATATATACATGATTTCTATCCCTGGAAAATTTGCATTAATGAAATAAAGCATTCTTAGTATAATAAGTCTGA

GCAGATGAGAATTTAAGTGAAGGTTCTATACACACCTGATGCACGACAGGCAGACCCCCAAATTGAGGCTTAGCCCGAGAG|GG|CGAG
                                                                                  >chr3
ATGGTAAATGTGATGTTTAGCACTGTGTTACATATATCATTACTCTGTTAATTCTTGCAGTAATTTAACTGGTCAGTAACAATAATAAT
:6928375-6928769 + E=1e-220
AATAATAATAATAATAAACAGTTTAATAAAATGCTTAGGATGTCCTAAGCACTTTGTATATATAATTCATTTAATCTATACTACACTAT

TTTATATAGATATTATCTTCCAAGTTTTTTTTCAGAGGACATTGAGGATCAGCAAGATTGAGTACTTTGCTTAACCCGTGAACTCAGGT

CAGAATTACTCCAAACATAATTTTTAACTACAAAATGTCTATCATTCTACCTCTGAATTTGTTGTTGTTTTTAACTTCCATACATCTGA

TTTGAAATTCCTTTATATATTTGGGGACCTT
```
